# Nationwide Analysis (2016-2020) of the Burden of Thrombocytopenia on Patients Admitted Due to Myocardial Infarction, Heart Failure or Atrial Fibrillation

**DOI:** 10.7759/cureus.78452

**Published:** 2025-02-03

**Authors:** Christian Siochi, Ben Lerman, Chioma Nwachukwu, Wilmer Cervantes, Bolaji Durodola, Lourdes Villarrubia Varela, Stephen Jesmajian

**Affiliations:** 1 Internal Medicine, Montefiore New Rochelle Hospital, Albert Einstein College of Medicine, New Rochelle, USA

**Keywords:** all cause mortality, atrial fibrillation, atrial flutter, heart failure, hospital resource utilization, inpatient mortality, length of hospital stay, myocardial infarction, national inpatient sample database, thrombocytopenia

## Abstract

Background: Myocardial infarction (MI), heart failure (HF) exacerbation, and atrial fibrillation/atrial flutter (AF) affect millions of patients every year, and thrombocytopenia is a common laboratory finding in hospitalized patients. This study aimed to investigate the impact of thrombocytopenia in patients admitted due to MI, HF, or AF in terms of mortality, length of stay, resource utilization, and need for intubation.

Methods: This is a National Inpatient Sample Database analysis from 2016-2020. Patients admitted with a primary diagnosis of MI, HF, or AF, with or without a secondary diagnosis of thrombocytopenia, were identified using ICD-10-CM codes. The primary outcome of our analysis was mortality. Secondary outcomes included length of stay, resource utilization, and necessity for endotracheal intubation. Univariate analysis was done for hospital-level and patient baseline characteristics such as age, gender, race, Charlson comorbidity index, insurance, hospital location, size, region, and teaching status. Baseline characteristics with p-value < 0.2 were considered significant and were adjusted in a multivariate analysis. Data was statistically significant if p-value <0.05.

Results: Among 3,093,479 patients who had a primary diagnosis of MI between 2016 and 2020, 5.13% (N=158,755) had thrombocytopenia. Among 1,187,164 patients who had a primary diagnosis of HF exacerbation, 5.92% (N=70,225) had thrombocytopenia. Of the 2,292,194 patients admitted due to AF, 3.53% (N=80,935) also had thrombocytopenia. Overall, outcomes were poor for all groups. Adjusted outcomes showed that in-hospital mortality was significantly higher in thrombocytopenic patients of all three groups: MI (OR 1.82; 95% CI 1.73 - 1.91; p<0.001), HF exacerbation (OR 2.13; 95% CI 1.96 - 2.32; p<0.001), AF (OR 2.29; 95% CI 2.02 - 2.6; p<0.001). Length of stay was significantly longer in thrombocytopenic patients of all three groups: MI (Regression coefficient 3.65; 95% CI 3.53 - 3.76; p<0.001), HF (Regression coefficient 2.39; 95% CI 2.19 - 2.59; p<0.001), AF (Regression coefficient 1.35; 95% CI 1.26 - 1.45; p<0.001). Resource utilization was significantly higher in thrombocytopenic patients of all three groups: MI (Regression coefficient 80,272.54; 95% CI 76,853.92 - 83,691.16; p<0.001), HF (Regression coefficient 40,802.52; 95% CI 36,367.31 - 45,237.74; p<0.001), AF (Regression coefficient 15,330.34; 95% CI 13,579.82 - 17,080.86; p<0.001). The need for endotracheal intubation was also increased in thrombocytopenic patients of all three groups: MI (OR 2.39; 95% CI 2.28 - 2.5; p<0.001), HF (OR 2.51; 95% CI 2.26 - 2.79; p<0.001), and AF (OR 2.88; 95% CI 2.53 - 3.28; p<0.001).

Conclusion: Thrombocytopenia has substantial negative outcomes in hospitalized patients with MI, HF, or AF. Thrombocytopenia could be a marker of poor prognosis, and greater awareness among physicians could potentially guide patient risk stratification, treatment decisions, resource allocation, and patient/ family counseling during hospitalization.

## Introduction

Thrombocytopenia, defined as platelet counts < 150x109/L, is a common hematologic abnormality seen in various medical conditions, including cardiovascular diseases (CVD). It can be sub-categorized as mild (100-149 × 10⁹/L), moderate (59-99 × 10⁹/L), and severe (<50 × 10⁹/L) [1].

Platelets are crucial for hemostasis, and their deficiency increases bleeding risk, while increased activity may lead to thrombus formation. Briefly, in some studies, low platelet count among hematologic indices has been associated with increased bleeding outcomes and mortality among patients with CVDs [2-4]. Conversely, other studies indicate an increased risk of all-cause mortality and myocardial infarction (MI) in patients with CVD and elevated platelet counts [5,6]. Platelets have thus been recognized as important factors in CVDs. Their dysregulation can lead to disruption of blood vessel integrity, thrombus formation, MI, and strokes [7]. Warkentin and Crowther (2009) proposed that preadmission low-normal platelet count, an important predisposing factor to in-hospital thrombocytopenia, may reflect a greater burden of atherosclerosis and/ or disease severity contributing to worsened cardiovascular outcomes. Also, medications such as heparin can trigger or worsen thrombocytopenia, and patients who develop heparin-induced thrombocytopenia can die as a direct consequence of thrombus formation, manifesting as acute cardiac ischemic events or heart failure [8]. Furthermore, studies that assess the impact of thrombocytopenia on AF and acute coronary syndrome showed that low platelet count was significantly associated with a higher risk of combined bleeding events culminating in increased mortality [8].

Thrombocytopenia is a frequent finding in hospitalized patients due to a combination of factors, such as patients’ underlying medical conditions and disease complications that can interfere with platelet synthesis or destruction, hemodynamic changes, and treatment strategies during hospitalization [9,10]. Patients with thrombocytopenia often experience longer hospital stays, greater resource utilization, and higher hospital costs, primarily due to delayed recovery from complications such as bleeding [11]. Thrombocytopenia in CVDs is commonly attributed to treatment with blood thinners (heparin, antiplatelets), diuretics for heart failure symptoms, or antidiabetic medications to manage diabetes, a common comorbidity [9,12]. Drug-induced thrombocytopenia is relatively common and potentially serious. Providers must remain vigilant and ready to discontinue or find alternatives to diuretics, antiplatelets, anticoagulants, and oral antidiabetic drugs that can induce thrombocytopenia and worsen outcomes in cardiac patients [13]. The prevalence and incidence of thrombocytopenia reported in patients with CVD varies according to the study methodology and the definition used. In patients with atrial fibrillation (AF), the prevalence of thrombocytopenia varies widely from 6-24 percent [14]. Data from the multicenter prospective cohort registry START (Survey on Anticoagulated Patients Register) showed that 592 of 5215 adult AF patients had documented thrombocytopenia [14]. Meanwhile, records from a single-center prospective cohort registry in Korea between 2000 and 2013 showed that 2656 of 10,978 adult patients with nonvalvular AF had low platelet counts [15]. An observational study suggested that baseline thrombocytopenia occurs in roughly 5% of patients with acute coronary syndrome (ACS), while incident thrombocytopenia can be seen in 13% of patients [3]. Among heart failure patients, the magnitude of thrombocytopenia has been recorded at 12.24%, reflecting its significant presence within this population [16].

Thrombocytopenia has been linked to negative clinical outcomes in several diseases. While this may be true for some diseases, there are conflicting data regarding platelet count and outcomes in common CVDs. There is a body of evidence documenting adverse outcomes in patients with concurrent thrombocytopenia and CVDs. Specifically, Polat et al. recorded one-year mortality in 29% of patients with heart failure (HF) and observed that platelet counts were significantly lower in this group [2]. Wang et al. reported an increased risk of bleeding and inpatient mortality among acute coronary syndrome patients with thrombocytopenia and demonstrated a direct correlation between the severity of thrombocytopenia and mortality risk [3]. Another study comparing bleeding rates in patients with concurrent AF and thrombocytopenia to those with normal platelet counts showed a higher one-year cumulative incidence of clinically relevant bleeding, vascular thrombotic events, and all-cause mortality among the thrombocytopenia group compared to controls [4]. In contrast to the above studies, high platelet counts and activation have rather been linked to poorer outcomes in patients with heart disease [17]. Bao et al. showed that an increased number of circulating platelets is associated with an increased risk of all-cause mortality in coronary artery disease patients with HF [5]. Similarly, a retrospective study on AF patients treated with different anticoagulants revealed higher rates of MI and mortality among patients with high platelet counts compared to normal [6].

CVDs remain the leading cause of death globally [18]. Coronary artery disease, heart failure (HF), and arrhythmias, especially AF, constitute a significant health burden and are among the most prevalent cardiac conditions in the population [18]. At present, the underlying mechanisms and the relationship between thrombocytopenia and MI/HF/AF outcomes remain unclear. With the existing knowledge gap and controversies in the literature, it is important to explore further the influence of platelet count on the outcomes of patients with AF, MI, and HF. Therefore, we conducted a retrospective cohort study to investigate a correlation in this population using discharge data from the National Inpatient Sample (NIS), Healthcare Cost and Utilization Project (HCUP), and Agency for Healthcare Research and Quality from 2016 to 2020.

The results of this study support that thrombocytopenia has a substantial negative impact on inpatient outcomes for patients admitted for CVD. Thrombocytopenic patients admitted for HF, MI, or AF not only had a higher mortality rate than those without thrombocytopenia but also had prolonged hospital stays, higher resource allocation, and cost burden, and increased chances of intubation. 

## Materials and methods

Study design 

This was a retrospective cohort study using discharge data from the National Inpatient Sample (NIS), Healthcare Cost and Utilization Project (HCUP), and Agency for Healthcare Research and Quality from 2016 to 2020. 

Study inclusion criteria 

Patients with an ICD-10-CM of MI, HF exacerbation, AF, or atrial flutter, aged >18 years, with or without a secondary diagnosis of thrombocytopenia were identified using the International Classification of Disease, Tenth Edition, Procedure Coding System (ICD-10-PCS) and Clinical Modification (ICD-10-CM) codes. 

Ethical considerations 

The data from the NIS-HCUP is publicly available, de-identified, and exempt from institutional review board approval. The need for informed consent was waived. 

Outcome measures 

The primary outcome of interest is in-hospital mortality, while secondary outcomes include length of stay, total hospital charges, and rate of endotracheal intubation; these secondary outcomes were assessed across all conditions. 

Statistical analysis 

This study used a confidence interval (CI) of 95% and a p-value <0.05 as statistically significant in its analysis. Continuous variables were examined through the calculation of means accompanied by standard deviations or medians along with interquartile ranges in the case of normally distributed and skewed data, respectively. Descriptive statistics incorporating frequencies and percentages were employed for the analysis of categorical variables. Patient and hospital-level baseline characteristics and in-hospital outcomes were compared between patients with a primary diagnosis of MI, HF, or AF, aged >18 years, with or without a secondary diagnosis of thrombocytopenia using the Pearson χ2 test for categorical variables and the independent sample t-test for continuous variables. Univariate and multivariate logistic regression were used to calculate unadjusted and adjusted odds ratios for in-hospital clinical outcomes. Univariate analysis was performed to identify which patient and hospital-level baseline characteristics were statistically significant with a p-value <0.2. These baseline characteristics were then used for adjustment in a multivariate analysis to account for potential confounding factors. All analyses were conducted using STATA v.13 (StataCorp, LLC, College Station, TX). 

## Results

Baseline patient characteristics, including race and Charlson comorbidity index, and hospital-level characteristics are shown in the following tables. In the United States, between 2016 and 2020, of the 3,093,479 patients with a primary diagnosis of MI, 5.13% (N=158,755) had thrombocytopenia, and 94.87% (N=2,934,724) did not. Among 1,187,164 patients who had a primary diagnosis of HF exacerbation, 5.92% (N=70,225) had thrombocytopenia and 94.08% (N=1,116,939) did not. Of the 2,292,194 patients admitted due to AF, 3.53% (N=80,935) had thrombocytopenia, and 96.47% (N=2,211,259) did not. 

The MI population with thrombocytopenia had a mean age of 69.74 years, while those without thrombocytopenia had a mean age of 66.62 years. The HF population with thrombocytopenia had a mean age of 72.21, while those without thrombocytopenia had a mean age of 70.46. The AF population with thrombocytopenia had a mean age of 71.67 years, while those without thrombocytopenia had a mean age of 70.56 years. 

The majority of patients included in this study were white, male, with a Charlson comorbidity index greater than 3. Tables 1-3 detail the percentage of patients with a primary diagnosis of MI, HF, or AF per baseline patient and hospital-level characteristics, with or without a secondary diagnosis of thrombocytopenia.

**Table 1:** Percentage of patients with a primary diagnosis of MI, per baseline patient and hospital-level characteristics, with or without a secondary diagnosis of thrombocytopenia. A multivariate regression model was later adjusted for patient and hospital-level baseline characteristics with a p-value <0.2 to account for potential confounding factors. MI: myocardial infarction

Baseline characteristics of MI patients		MI patients without thrombocytopenia		MI patients with thrombocytopenia		Total MI patients		p-value
		%	N	%	N	%	N	
Sex	Male	62.52	1,834,789.445	69.7	110,652.235	62.89	1,945,488.943	<0.001
	Female	37.48	1,099,934.555	30.3	48,102.765	37.11	1,147,990.057	
Race	White	73.63	2,160,837.281	72.5	115,097.375	73.57	2,275,872.5	<0.001
	Black	11.28	331,036.8672	10.55	16,748.6525	11.25	348,016.3875	
	Hispanic	8.73	256,201.4052	9.14	14,510.207	8.75	270,679.4125	
	Asian	2.74	80,411.4376	4.12	6,540.706	2.81	86,926.7599	
	Native American	0.59	17,314.8716	0.6	952.53	0.59	18,251.5261	
	Other	3.03	88,922.1372	3.1	4,921.405	3.03	93,732.4137	
Charlson comorbidity index	0	0	0	0	0	0	0	<0.001
	1	24.35	714,605.294	10.94	17,367.797	23.66	731,917.1314	
	2	25.1	736,615.724	18.88	29,972.944	24.78	766,564.0962	
	> 3	50.55	1,483,502.982	70.17	111,398.3835	51.56	1,594,997.772	
Median income	<49,999	30.69	900,666.7956	29.14	46,261.207	30.61	946,913.9219	<0.001
	50,000-64,999	27.7	812,918.548	26.82	42,578.091	27.66	855,656.2914	
	65,000-85,999	23.39	686,431.9436	24.15	38,339.3325	23.43	724,802.1297	
	>86,000	18.21	534,413.2404	19.9	31,592.245	18.3	566,106.657	
Insurance type	Medicare	58	1,702,139.92	68.18	108,239.159	58.52	1,810,303.911	<0.001
	Medicaid	9.89	290,244.2036	8	12,700.4	9.8	303,160.942	
	Private insurance	27.12	795,897.1488	20.59	32,687.6545	26.79	828,743.0241	
	Self Pay	4.98	146,149.2552	3.23	5,127.7865	4.89	151,271.1231	
Hospital region	Northeast	17.39	510,348.5036	15.09	23,956.1295	17.27	534,243.8233	<0.001
	Midwest	22.3	654,443.452	23.9	37,942.445	22.38	692,320.6002	
	South	41.38	1,214,388.791	39.7	63,025.735	41.29	1,277,297.479	
	West	18.94	555,836.7256	21.31	33,830.6905	19.06	589,617.0974	
Hospital bed size	Small	18.27	536,174.0748	14.51	23,035.3505	18.08	559,301.0032	<0.001
	Medium	30.47	894,210.4028	28.87	45,832.5685	30.39	940,108.2681	
	Large	51.26	1,504,339.522	56.62	89,887.081	51.54	1,594,379.077	
Hospital location	Rural	7.7	225,973.748	4.89	7,763.1195	7.55	233,557.6645	<0.001
	Urban	92.3	2,708,750.252	95.11	150,991.8805	92.45	2,859,921.336	
Hospital teaching status	No	30.85	905,362.354	24.14	38323.457	30.5	943,511.095	<0.001
	Yes	69.15	2,029,361.646	75.86	120431.543	69.5	2,149,967.905	

**Table 2:** Percentage of patients with a primary diagnosis of HF exacerbation, per baseline patient and hospital-level characteristics, with or without a secondary diagnosis of thrombocytopenia. A multivariate regression model was later adjusted for patient and hospital-level baseline characteristics with a p-value <0.2 to account for potential confounding factors. HF: heart failure

Baseline characteristics of HF patients		HF patients without thrombocytopenia		HF patients with thrombocytopenia		Total HF patients		p-value
		%	N	%	N	%	N	
Sex	Male	52.01	580,919.9739	61.29	43,040.9025	52.55	623,854.682	<0.001
	Female	47.99	536,019.0261	38.71	27,184.0975	47.45	563,309.318	
Race	White	70.43	786,660.1377	71.29	50,063.4025	70.48	836,713.1872	<0.001
	Black	17.57	196,246.1823	15.06	10,575.885	17.43	206,922.6852	
	Hispanic	7.21	80,531.3019	7.66	5,379.235	7.24	85,950.6736	
	Asian	1.99	22,227.0861	3.02	2,120.795	2.05	24,336.862	
	Native American	0.59	6,589.9401	0.61	428.3725	0.59	7,004.2676	
	Other	2.2	24572.658	2.35	1650.2875	2.21	26236.3244	
Charlson comorbidity index	0	0	0	0	0	0	0	<0.001
	1	13.29	148,441.1931	8.62	6,053.395	13.02	154,568.7528	
	2	20.73	231,541.4547	14.83	10,414.3675	20.38	241,944.0232	
	> 3	65.97	736,844.6583	76.55	53,757.2375	66.6	790,651.224	
Median income	<49,999	32.69	365,127.3591	29.38	20,632.105	32.5	385,828.3	<0.001
	50,000-64,999	26.93	300,791.6727	26.09	18,321.7025	26.88	319,109.6832	
	65,000-85,999	23.07	257,677.8273	23.78	16,699.505	23.12	274,472.3168	
	>86,000	17.3	193,230.447	20.76	14,578.71	17.51	207,872.4164	
Insurance type	Medicare	71.77	801,627.1203	75.62	53,104.145	72	854,758.08	<0.001
	Medicaid	12.14	135,596.3946	10.09	7,085.7025	12.02	142,697.1128	
	Private insurance	12.94	144,531.9066	12.11	8,504.2475	12.89	153,025.4396	
	Self pay	3.15	35,183.5785	2.18	1,530.905	3.09	36,683.3676	
Hospital region	Northeast	19.38	216,462.7782	18.06	12,682.635	19.3	229,122.652	<0.001
	Midwest	23.31	260,358.4809	22.94	16,109.615	23.29	276,490.4956	
	South	38.3	427,787.637	36.77	25,821.7325	38.21	453,615.3644	
	West	19.01	212,330.1039	22.24	15,618.04	19.2	227,935.488	
Hospital bed size	Small	21.39	238,913.2521	18.45	12,956.5125	21.22	251,916.2008	<0.001
	Medium	28.64	319,891.3296	27.73	19,473.3925	28.59	339,410.1876	
	Large	49.96	558,022.7244	53.82	37,795.095	50.19	595,837.6116	
Hospital location	Rural	12.7	141,851.253	9.32	6,544.97	12.5	148,395.5	<0.001
	Urban	87.3	975,087.747	90.68	63,680.03	87.5	1,038,768.5	
Hospital teaching status	No	38.68	432,032.0052	33.07	23,223.4075	38.35	455,277.394	<0.001
	Yes	61.32	684,906.9948	66.93	47,001.5925	61.65	731,886.606	

**Table 3:** Percentage of patients with a primary diagnosis of AF, per baseline patient and hospital-level characteristics, with or without a secondary diagnosis of thrombocytopenia. A multivariate regression model was later adjusted for patient and hospital-level baseline characteristics with a p-value <0.2 to account for potential confounding factors. AF: atrial fibrillation/atrial flutter

Baseline characteristics of AF patients		AF patients without thrombocytopenia		AF patients with thrombocytopenia		Total AF patients		p-value
		%	N	%	N	%	N	
Sex	Male	50.61	1,119,118.18	62.49	50,576.2815	51.03	1,169,706.598	<0.001
	Female	49.39	1,092,140.82	37.51	30,358.7185	48.97	1,122,487.402	
Race	White	81.85	1,809,915.492	78.63	63,639.1905	81.74	1,873,639.376	<0.001
	Black	8.42	186,188.0078	9.71	7,858.7885	8.46	193,919.6124	
	Hispanic	5.82	128,695.2738	6.9	5,584.515	5.85	134,093.349	
	Asian	1.48	32,726.6332	2.27	1,837.2245	1.51	34,612.1294	
	Native American	0.37	8,181.6583	0.47	380.3945	0.37	8,481.1178	
	Other	2.06	45,551.9354	2.03	1,642.9805	2.06	47,219.1964	
Charlson comorbidity index	0	22.66	501,071.2894	10.8	8,740.98	22.24	509,783.9456	<0.001
	1	25.83	571,168.1997	18.11	14,657.3285	25.56	585,884.7864	
	2	19.64	434,291.2676	19.32	15,636.642	19.63	449,957.6822	
	> 3	31.87	704,728.2433	51.77	41,900.0495	32.57	746,567.5858	
Median income	<49,999	27.42	606,327.2178	27.88	22,564.678	27.44	628,978.0336	<0.001
	50,000-64,999	27.34	604,558.2106	26.22	21,221.157	27.3	625,768.962	
	65,000-85,999	24.71	546,402.0989	24.41	19,756.2335	24.7	566,171.918	
	>86,000	20.53	453,971.4727	21.49	17,392.9315	20.56	471,275.0864	
Insurance type	Medicare	70.1	1,550,092.559	74.35	60,175.1725	70.25	1,610,266.285	<0.001
	Medicaid	6.36	140,636.0724	8.17	6,612.3895	6.42	147,158.8548	
	Private insurance	21.06	465,691.1454	14.92	12,075.502	20.84	477,693.2296	
	Self Pay	2.48	54,839.2232	2.56	2,071.936	2.49	57,075.6306	
Hospital region	Northeast	19.66	434,733.5194	17.87	14,463.0845	19.6	449,270.024	<0.001
	Midwest	24.24	536,009.1816	23.74	19,213.969	24.22	555,169.3868	
	South	40.93	905,068.3087	39.87	32,268.7845	40.89	937,278.1266	
	West	15.18	335,669.1162	18.53	14,997.2555	15.29	350,476.4626	
Hospital bed size	Small	21.09	466,354.5231	20.02	16,203.187	21.05	482,506.837	<0.001
	Medium	30.01	663,598.8259	29.23	23,657.3005	29.98	687,199.7612	
	Large	48.9	1,081,305.651	50.76	41,082.606	48.97	1,122,487.402	
Hospital location	Rural	10.66	235,720.2094	8.14	6,588.109	10.58	242,514.1252	<0.001
	Urban	89.34	1,975,538.791	91.86	74,346.891	89.42	2,049,679.875	
Hospital teaching status	No	34.22	756,692.8298	32.45	26,263.4075	34.16	783,013.4704	<0.001
	Yes	65.78	1,454,566.17	67.55	54,671.5925	65.84	1,509,180.53	

Primary outcomes 

Unadjusted outcomes showed that in-hospital mortality was significantly higher in thrombocytopenic patients of all three groups: MI (OR 2.29; 95% (CI 2.2 - 2.38; p<0.001)), HF exacerbation (OR 2.39; 95% (CI 2.22 - 2.58; p<0.001)), AF (OR 3.25; 95% (CI 2.93 - 3.6; p<0.00)) (Table 4). 

**Table 4:** Using non-thrombocytopenic patients as a reference, unadjusted odds ratio for in-hospital mortality outcome of patients admitted for MI, HF, AF, with a secondary diagnosis of thrombocytopenia. This study used a confidence interval of 95% and a p-value <0.05 as statistically significant in its analysis. MI: myocardial infarction; HF: heart failure exacerbation; AF: atrial fibrillation/atrial flutter

In-patient mortality	Odds Ratio	Std Error	t	p-value	95% CI	
MI	2.29	0.05	39.17	<0.001	2.2	2.38
HF	2.39	0.09	22.99	<0.001	2.22	2.58
AF	3.25	0.17	22.35	<0.001	2.93	3.6

Adjusted outcomes showed that in-hospital mortality was significantly higher in thrombocytopenic patients of all three groups: MI (OR 1.82; 95% (CI 1.73 - 1.91; p<0.001)), HF exacerbation (OR 2.13; 95% (CI 1.96 - 2.32; p<0.001)), AF (OR 2.29; 95% (CI 2.02 - 2.6; p<0.001)) (Table 5). 

**Table 5:** Using non-thrombocytopenic patients as a reference, adjusted odds ratio for in-hospital mortality outcome of patients admitted for MI, HF, AF, with a secondary diagnosis of thrombocytopenia. This study used a confidence interval of 95% and a p-value <0.05 as statistically significant in its analysis. MI: myocardial infarction; HF: heart failure exacerbation; AF: atrial fibrillation/atrial flutter

In-patient mortality	Odds ratio	Std error	t	p-value	95% CI	
MI	1.82	0.05	23.3	<0.001	1.73	1.91
HF	2.13	0.09	17.76	<0.001	1.96	2.32
AF	2.29	0.15	12.92	<0.001	2.02	2.6

Secondary outcomes 

As shown in Table 6, unadjusted outcomes revealed that length of stay was significantly longer in thrombocytopenic patients of all three groups: MI (Regression coefficient 4.39; 95% (CI 4.28 - 4.49; p<0.001)), HF (Regression coefficient 2.68; 95% (CI 2.48 - 2.88; p<0.001)), AF (Regression coefficient 1.78; 95% (CI 1.7 - 1.87; p<0.001)). Resource utilization was also significantly higher in thrombocytopenic patients of all three groups: MI (Regression coefficient 93,858.91; 95% (CI 90,344.02 - 97,373.8; p<0.001)), HF (Regression coefficient 48,407.85; 95% (CI 42,462.45 - 54,353.24; p<0.001)), AF (Regression coefficient 20,921.81; 95% (CI 19,316.12 - 22,527.5; p<0.001)). 

**Table 6:** Unadjusted regression coefficient for length of stay and total hospital charges outcomes of patients admitted for MI, HF, AF with a secondary diagnosis of thrombocytopenia. This study used a confidence interval of 95% and a p-value <0.05 as statistically significant in its analysis. MI: myocardial infarction; HF: heart failure exacerbation; AF: atrial fibrillation/atrial flutter

Outcome	Coefficient	Std Error	t	p-value	95% CI	
Length of stay (days)						
MI	4.39	0.05	83.47	<0.001	4.28	4.49
HF	2.68	0.1	26.44	<0.001	2.48	2.88
AF	1.78	0.04	39.88	<0.001	1.7	1.87
Total hospital charges ($)						
MI	93,858.91	1,793.23	52.34	<0.001	90,344.02	97,373.8
HF	48,407.85	3,033.23	15.96	<0.001	42,462.45	54,353.24
AF	20,921.81	819.19	25.54	<0.001	19,316.12	22,527.5

Table 7 details adjusted outcomes showing that length of stay was significantly longer in thrombocytopenic patients of all three groups: MI (Regression coefficient 3.65; 95% (CI 3.53 - 3.76; p<0.001)), HF (Regression coefficient 2.39; 95% (CI 2.19 - 2.59; p<0.001)), AF (Regression coefficient 1.35; 95% (CI 1.26 - 1.45; p<0.001)). Adjusted outcomes also revealed that resource utilization was significantly higher in thrombocytopenic patients of all three groups: MI (Regression coefficient 80,272.54; 95% (CI 76,853.92 - 83,691.16; p<0.001)), HF (Regression coefficient 40,802.52; 95% (CI 36,367.31 - 45,237.74; p<0.001)), AF (Regression coefficient 15330.34; 95% (CI 13,579.82 - 17,080.86; p<0.001)).

**Table 7:** Adjusted regression coefficient for length of stay and total hospital charges outcomes of patients admitted for MI, HF, AF, with a secondary diagnosis of thrombocytopenia. This study used a confidence interval of 95% and a p-value <0.05 as statistically significant in its analysis. MI: myocardial infarction; HF: heart failure exacerbation; AF: atrial fibrillation/atrial flutter

Outcome	Coefficient	Std Error	t	p-value	95% CI	
Length of stay (days)						
MI	3.65	0.06	61.72	<0.001	3.53	3.76
HF	2.39	0.1	23.75	<0.001	2.19	2.59
AF	1.35	0.05	27.61	<0.001	1.26	1.45
Total hospital charges ($)						
MI	80,272.54	1,744.12	46.02	<0.001	76,853.92	83,691.16
HF	40,802.52	2,262.77	18.03	<0.001	36,367.31	45,237.74
AF	15,330.34	893.08	17.17	<0.001	13,579.82	17,080.86

Unadjusted outcomes showed that the need for endotracheal intubation was increased in thrombocytopenic patients of all three groups: MI (OR 2.86; 95% (CI 2.75 - 2.98; p<0.001)), HF (OR 2.7; 95% (CI 2.45 - 2.97; p<0.001)), and AF (OR 3.94; 95% (CI 3.54 - 4.39; p<0.001)) (Table 8).

**Table 8:** Unadjusted odds ratio for endotracheal intubation outcomes of patients admitted for MI, HF, or AF with a secondary diagnosis of thrombocytopenia. MI: myocardial infarction; HF: heart failure exacerbation; AF: atrial fibrillation/atrial flutter This study used a confidence interval (CI) of 95% and a p-value <0.05 as statistically significant in its analysis.

Endotracheal intubation	Odds Ratio	Std Error	t	p-value	95% CI	
MI	2.86	0.06	50.63	<0.001	2.75	2.98
HF	2.7	0.13	20.48	<0.001	2.45	2.97
AF	3.94	0.22	25.17	<0.001	3.54	4.39

Adjusted outcomes also noted that the need for endotracheal intubation was increased in thrombocytopenic patients of all three groups: MI (OR 2.39; 95% (CI 2.28 - 2.5; p<0.001)), HF (OR 2.51; 95% (CI 2.26 - 2.79; p<0.001)), and AF (OR 2.88; 95% (CI 2.53 - 3.28; p<0.001)) (Table 9). 

**Table 9:** Adjusted odds ratio for endotracheal intubation outcomes of patients admitted for MI, HF, or AF with a secondary diagnosis of thrombocytopenia. MI: myocardial infarction; HF: heart failure exacerbation; AF: atrial fibrillation/atrial flutter This study used a confidence interval (CI) of 95% and a p-value <0.05 as statistically significant in its analysis.

Endotracheal intubation	Odds Ratio	Std Error	t	p-value	95% CI	
MI	2.39	0.06	35.71	<0.001	2.28	2.5
HF	2.51	0.14	17.12	<0.001	2.26	2.79
AF	2.88	0.19	16.06	<0.001	2.53	3.28

## Discussion

Thrombocytopenia and AF

It is known that one of the major treatment components for patients with AF is anticoagulation therapy [9]. This inherently carries the risk of bleeding, which is compounded by concurrent thrombocytopenia. As such, it is highly plausible that bleeding could contribute to the increased mortality in this group. This is supported by the findings in the AFIRE study, which showed increased bleeding events in patients with AF and thrombocytopenia [19]. Data comparing different anticoagulation therapies is scarce; however, just as in a normal population, thrombocytopenic patients tend to have lower bleeding rates on direct oral anticoagulants (DOAC) than warfarin [20]. Furthermore, bleeding events, regardless of severity, often lead to lengthy and costly hospital stays, especially if they require blood transfusions. 

Thrombocytopenia and HF

There has historically been a link between platelet function and HF. Emerging data observe negative outcomes in patients with thrombocytopenia and HF. It has been shown that P-selectin, a pro-inflammatory cell adhesion molecule expressed on the surface of activated platelets, is higher in patients with acute HF compared to stable HF [21]. Higher P-selectin levels potentially exacerbate inflammatory responses, contributing to vascular dysfunction, tissue injury, and overall worse clinical outcomes. Many HF medications, including diuretics, beta-blockers, and angiotensin-converting enzyme inhibitors/angiotensin receptor blockers (ACEi/ARB), can have deleterious effects on platelet function [22]. One possible theory is that patients with thrombocytopenia may experience further impairment of their platelet function due to HF treatment. This is particularly important as thrombocytopenia has been associated with worsening disease severity [22]. 

It was reported that patients with decompensated HF have an increased mean platelet volume compared to those with stable HF [21]. High platelet volume is thought to be an indicator of platelet activation and a marker of HF severity. It is possible that the negative outcome in patients with concomitant HF and thrombocytopenia is due to accelerated platelet activation and destruction in the disease course [21]. 

Thrombocytopenia and MI

Poor outcomes were observed in patients with concurrent thrombocytopenia and MI, like the AF cohort, potentially linked to elevated bleeding risks. Treatment decisions in patients with MI and thrombocytopenia are carefully considered, especially as it pertains to antiplatelet therapy. Eikelboom et al. identified thrombocytopenia as a strong predictor of major bleeding during ACS hospitalization [23]. This may explain why patients are less likely to receive drug-eluting stents that require some antiplatelet therapy for a specified period. Relatedly, the initial treatment for MI and ACS typically involves heparin therapy. However, treatment with heparin can result in heparin-induced thrombocytopenia, a clinical condition that paradoxically increases thrombosis risk and potentially worsens outcomes [23]. While treatment for HIT is to stop the heparin, if anticoagulation is still necessary, it is possible to use a different anticoagulant, such as a DOAC [13].

Thrombocytopenia and CVD 

Patients with thrombocytopenia often have concurrent anemia and are commonly given both red blood cells and platelet transfusions, even without a bleeding source being identified. These blood products (especially platelets) are pro-inflammatory and can worsen outcomes in already-susceptible patients [24,25]. Their role in the pro-inflammatory process has been linked to leukocyte recruitment [26]. While increased bleeding risk is an inherent risk in thrombocytopenic patients, there are other possible links between CVD and reduced platelet count that lead to poor outcomes. Platelets play an important role in endothelial function by preventing vascular leakage. Platelet dysfunction is linked to atherogenesis via the NO and PGI2 pathways. Since endothelial dysfunction is a major component of CVD, any platelet dysfunction will likely impact the endothelium, leading to worse outcomes in patients who are already vulnerable to cardiovascular complications [26]. 

Besides their role in hemostasis, platelets have been shown to play relevant roles in response to ongoing infection through immune cell translocation and cytokine release. It is, therefore, logical that low platelet count may contribute to infection propagation, especially in the hospital setting [27]. Wang et al. documented a remarkable increase in infection rate and major adverse cardiac events among hospitalized STEMI patients with thrombocytopenia [27]. Finally, patients with thrombocytopenia have multiple comorbidities, including but not limited to chronic kidney disease and chronic obstructive pulmonary disease, that are shared across patients with CVDs [28]. Patients with additional comorbidities have a higher likelihood of prolonged hospitalization, development of complications, higher chances of adverse events, and overall worse outcomes. 

Thrombocytopenia and resource utilization

The data regarding overall thrombocytopenia and healthcare resource utilization is sparse. With regards to our outcomes, it is seemingly limited to a study that showed that thrombocytopenia and PCI of complete total occlusion had higher resource use and worse outcomes [29]. Our data shows that thrombocytopenia increased resource utilization and length of stay across all outcomes. As there is increasing strain on healthcare systems, decreasing length of stay and hospital costs have become more important, as this allows for more bed availability and the ability for hospitals to spread resources. Furthermore, shorter length of stay has favorable post-discharge outcomes [29].

Study limitations and future directions 

There is an association between thrombocytopenia and various CVDs. This is supported by both in vitro and in vivo studies. However, the underlying mechanisms mediating this association remain under investigation. 

We did not have access to readmission data; however, in the future, we intend to incorporate the same patients’ data through multiple admissions. This is feasible as diseases such as HF, which is a leading cause of readmission in older adults [30]. This study is also limited by coding variability. The National Inpatient Sample is a repository of hospital administrative data; any extrapolation is subject to misclassification in coding, as well as variability in definitions. For example, a patient may have a suspected MI which was subsequently ruled out, but the coding for MI may have remained throughout their stay.

Clearly, our study is limited by the same constraints linked to retrospective observational investigations. Lack of randomization leads to difficulty ascertaining which and if specific factors contribute to certain outcomes. Further research can include not only randomized control trials as well as more longitudinal data incorporating more follow-up. The database also lacks information on medications, which means we are only able to speculate whether thrombocytopenia is related to medication. For example, we do not know if a patient was being treated for AF with a DOAC, warfarin, or no anticoagulants at all, which would affect bleeding risk and subsequent treatment. The same example applies to HF and diuretics. Furthermore, this study compiles data from across the country; it assumes that there is a standard diagnostic and treatment strategy. Moreover, the data do not include any follow-up data, confining outcomes to in-hospital only. 

Finally, patients with thrombocytopenia are often excluded from clinical trials, the cornerstone for scientific research and knowledge, as it is considered a confounder. Specific trials designed around thrombocytopenic patients can help shed light on treatment strategies for this population. As such, additional prospective studies should be conducted to both address these limitations and further enhance the depth of knowledge regarding why and how thrombocytopenia affects patients with AF, HF, and MI.

## Conclusions

The prevalence of thrombocytopenia and AF, HF, and MI (both concurrent and independent) highlight the importance of our research in the outcomes of these patients. This investigation revealed that thrombocytopenia had a significant impact on patients hospitalized for AF, HF, or MI. This includes higher mortality rates and increased likelihood of intubation, as well as length and cost of stay. These findings underscore the importance of continued investigations regarding the etiology and pathophysiology behind thrombocytopenia and cardiovascular disease. Additional prospective cohort studies are needed to explore the effects of thrombocytopenia on the AF, HF, and MI populations. 
